# Clinical Outcomes of Ruxolitinib Treatment in Patients With IPSS Intermediate‐1‐Risk Myelofibrosis: Interim Analysis From an Italian, Prospective Study (ROMEI)

**DOI:** 10.1002/hon.70178

**Published:** 2026-03-25

**Authors:** Paola Guglielmelli, Massimo Breccia, Francesco Mendicino, Maurizio Martelli, Nicola Di Renzo, Giuseppe A. Palumbo, Monica Crugnola, Maurizio Musso, Silvia Sibilla, Paolo Sportoletti, Elisabetta Abruzzese, Stefana Impera, Alessandra Malato, Sergio Siragusa, Carmine Selleri, Fabrizio Pane, Bruno Martino, Alessandra Ricco, Angelo Gardellini, Anna Marina Liberati, Agostino Tafuri, Maria Langella, Marco Brociner, Paolo Ditonno, Domenico Pastore, Olga Mulas, Barbara Pocali, Marco De Gobbi, Erika Morsia, Giulia Benevolo, Elena Maria Elli, Valerio De Stefano, Antonietta Pia Falcone, Daniele Vallisa, Simona Tomassetti, Francesca Lunghi, Nicola Orofino, Giuseppe Carli, Tiziana Urbano, Alessandro Lucchesi, Marta Brunoventre, Massimiliano Bonifacio, Gianni Binotto, Francesco Cavazzini, Paola Ranalli, Alessandro Allegra, Barbara Anaclerico, Serena Mazzotta, Filippo Gherlinzoni, Mario Tiribelli, Chiara Castiglioni, Marina Landoni, Diletta Valsecchi, Michela Magnoli, Francesco Passamonti, Francesca Palandri

**Affiliations:** ^1^ Department of Experimental and Clinical Medicine CRIMM Center for Research and Innovation of Myeloproliferative Neoplasms AOU Careggi University of Florence Florence Italy; ^2^ Hematology Department of Translational and Precision Medicine Azienda Policlinico Umberto I Sapienza University Rome Italy; ^3^ A. O. di Cosenza Presidio Ospedaliero Annunziata Cosenza Italy; ^4^ A. O. Policlinico Umberto I Università La Sapienza Roma Italy; ^5^ Hematology and Hematopoietic Stem Cell Transplantation Unit. PO “Vito Fazzi”. ASL Lecce Lecce Italy; ^6^ Department of Scienze Mediche Chirurgiche e Tecnologie Avanzate “G.F. Ingrassia” University of Catania Catania Italy; ^7^ Hematology Unit University Hospital of Parma Parma Italy; ^8^ Ospedale La Maddalena Palermo Italy; ^9^ Unit of Hematology and Hemopoietic Stem Cell Transplantation Ospedale Cardinale G Panico Tricase Italy; ^10^ Institute of Hematology‐Centro di Ricerche Emato‐Oncologiche (CREO) University of Perugia Perugia Italy; ^11^ Hematology S. Eugenio Hopsital ASL Roma 2 Tor Vergata University Rome Italy; ^12^ U.O.C. Ematologia A. O.ad Alta Specializzazione Ospedale Garibaldi Nesima Catania Italy; ^13^ A.O. Ospedali Riuniti Villa Sofia‐Cervello ‐ P.O. Cervello Palermo Italy; ^14^ Az. Osp. Univ. Policlinico P Giaccone Università degli Studi Palermo Palermo Italy; ^15^ Hematology and Bone Marrow Unit AOU S.Giovanni di Dio e Ruggi d'Aragona Department of Medicine and Surgery University of Salerno Fisciano Italy; ^16^ A. O. U. Università degli Studi della Campania Luigi Vanvitelli Napoli Italy; ^17^ A.O. Bianchi Melacrino Morelli ‐ Presidio Ospedali Riuniti Reggio di Calabria Italy; ^18^ Az. Osp. Univ. Consorziale Policlinico di Bari Università degli Studi Bari Italy; ^19^ Division of Hematology Valduce Hospital Como Italy; ^20^ S.C. Oncoematologia Azienda Ospedaliera S Maria di Terni Terni Italy; ^21^ Hematology Department of Clinical and Molecular Medicine University Hospital Sant'Andrea‐Sapienza Rome Italy; ^22^ Presidio Ospedaliero Andrea Tortora ‐ ASL Salerno Pagani Italy; ^23^ ASST Sette Laghi Osp. di Circolo e Fond Macchi Varese Italy; ^24^ IRCCS Istituto Tumori “Giovanni Paolo II” Bari Italy; ^25^ UOC Ematologia e Trapianto di Midollo, Presidio Osp. A. Perrino ASL BR Brindisi Italy; ^26^ A. O. Brotzu Osp. Onc. Armando Businco Centro Rif Onc. Reg Cagliari Italy; ^27^ Dip. Pneumo‐Oncoematologico Azienda Ospedaliera di Rilievo Nazionale A. Cardarelli Napoli Italy; ^28^ Department Clinical and Biological Sciences University of Torino A.O.U. S. Luigi Gonzaga Orbassano Turin Italy; ^29^ Department of Hematology A.O.U. Ospedali Riuniti di Ancona Presidio Umberto I Ancona Italy; ^30^ Hematology U., A.O.U. Città della Salute e della Scienza Turin Italy; ^31^ Hematology Division and Bone Marrow Unit Fondazione IRCCS San Gerardo dei Tintori Monza Italy; ^32^ Section of Hematology Catholic University Fondazione Policlinico A. Gemelli IRCCS Roma Italy; ^33^ Division of Hematology IRCCS Casa Sollievo della Sofferenza San Giovanni Rotondo Italy; ^34^ Hematology and Bone Marrow Transplant Unit Presidio Ospedaliero di Piacenza AUSL Piacenza Italy; ^35^ Hematology Unit Infermi Hospital Rimini Rimini Italy; ^36^ Unit of Hematology and Bone Marrow Transplantation IRCCS San Raffaele Hospital Milan Italy; ^37^ Division of Hematology Legnano's Hospital Milan Italy; ^38^ Presidio Ospedaliero S. Bortolo ULSS 6 Vicenza Vicenza Italy; ^39^ Hematology Ospedale San Giuseppe Moscati Taranto Italy; ^40^ IRCCS Istituto Romagnolo per lo Studio dei Tumori (IRST) ‘Dino Amadori’ Meldola Italy; ^41^ U.O. Oncologia Medica ASST Fatebenefratelli Sacco Ospedale Fatebenefratelli Oftalmico Milano Italy; ^42^ Department of Engineering for Innovation Medicine Section of Innovation Biomedicine University of Verona Verona Italy; ^43^ Department of Medicine Hematology Unit Padova University Hospital Padova Italy; ^44^ Az. Osp. Univ. Ferrara Arcispedale S Anna Univ. degli Studi Ferrara Italy; ^45^ Department of Medicine and Aging Sciences “G. D'Annunzio” University Chieti Italy; ^46^ Hematology Unit Department of Oncology and Hematology Ospedale Civile “Santo Spirito” Pescara Italy; ^47^ Az. Ospe.Universitaria Policlinico G. Martino Univ. di Messina Italy; ^48^ Azienda Ospedaliera S Giovanni Addolorata Rome Italy; ^49^ Division of Hematology Ospedale “C. e G. Mazzoni” ASUR Marche‐AV5 Ascoli Piceno Italy; ^50^ Pres.Ospedaliero S. Maria di Ca’ Foncello Treviso Italy; ^51^ Division of Hematology and BMT ASUFC and Department of Medicine University of Udine Udine Italy; ^52^ Novartis Farma S.P.A. Milano Italy; ^53^ OPIS s.r.l. Desio Italy; ^54^ Università degli Studi di Milano Fondazione IRCCS Ca’ Granda Ospedale Maggiore Policlinico Milan Italy; ^55^ IRCCS Azienda Ospedaliero‐Universitaria di Bologna Istituto di Ematologia “Seràgnoli” Bologna Italy

**Keywords:** efficacy, intermediate‐1 IPSS, myelofibrosis, ROMEI study, ruxolitinib, safety

## Abstract

Ruxolitinib (RUX), a JAK1/2 inhibitor, demonstrated treatment benefits for myelofibrosis (MF) in intermediate‐1 (Int‐1)–risk patients with a significant disease burden; however, the evidence is scarce. This interim analysis investigated the efficacy and safety of ruxolitinib in patients with Int‐1‐risk MF. ROMEI, a multicenter, observational, prospective study, enrolled 508 adult patients with MF receiving ruxolitinib according to approved indications. The present interim analysis was focused on 107 eligible patients in the Int‐1‐risk group. Primary endpoints included changes in symptoms response and health‐related quality of life scores. Secondary endpoints included spleen response evaluation, overall survival, and safety including dosing pattern and dose interruptions. Among the 107 Int‐1‐risk patients with a median age of 63 years, 65.5% were highly symptomatic (total symptoms score: ≥ 20), while the spleen was palpable at ≥ 5 cm and ≥ 10 cm in 74% and 27% of patients, respectively, with baseline EuroQol visual analogue scale (EQ‐VAS) score of 65.1 ± 19.4. After RUX treatment, 42.1% and 43.9% of patients demonstrated a symptom response at 24 and 48 weeks, while 38.9% and 46.8% showed a spleen response at 24 and 48 weeks, respectively. EQ‐VAS increased to 71.8 ± 16.3 at 24 weeks and 69.3 ± 19.2 at 48 weeks. Furthermore, 11.2% and 25.2% of patients reported temporary and permanent discontinuation, respectively with no new adverse events reported. The interim analysis showed that ruxolitinib provided clinical benefits, a manageable safety profile, and improved quality of life for Int‐1‐risk subgroup patients with frequent and sustained responses with acceptable toxicity.

## 1 Introduction

Myelofibrosis (MF) is a rare, chronic, Philadelphia chromosome‐negative myeloproliferative neoplasm (MPN) primarily characterized by debilitating constitutional symptoms and clinical signs including marked splenomegaly and bone marrow fibrosis, all which impact quality of life (QoL) and reduce survival [1, 2].

Ruxolitinib, the first oral selective JAK1/JAK2 inhibitor, has demonstrated durable improvements in splenomegaly and MF‐related symptoms and a positive impact on overall survival (OS), regardless of driver mutations, in both clinical trials and clinical practice settings, with a manageable safety profile [3, 4, 5]. The COMFORT efficacy trials, leading to the registration of ruxolitinib and its use in MF [4], did not include patients with intermediate‐1 (Int‐1)–risk MF. The JUMP trial documented the efficacy of ruxolitinib in this patient population [6, 7, 8]. Despite being classified as having a lower risk, patients with International Prognostic Scoring System (IPSS) Int‐1‐risk may have a significant symptom burden and splenomegaly correlating negatively with OS, thus requiring timely treatment [9]. Recently, ruxolitinib therapy in Int‐1‐risk patients has led to better event‐free survival and OS and lower treatment discontinuation in this patient population than in higher risk patients [10]. Nonetheless, literature describing ruxolitinib impact in the lower risk categories is limited [10, 11].

ROMEI (Ruxolitinib Observational study in Myelofibrosis Treated PatiEnts in Italy; (CINC424AIT04), an ongoing, Novartis‐sponsored, Italian, prospective, clinical study, enrolled patients who received ruxolitinib according to approved indications [12, 13]. Early results from the ROMEI study established the beneficial effect of ruxolitinib in reducing spleen size and improving symptoms and QoL, with a favorable safety profile, consistent with previous findings [12, 13].

The main objectives of this interim analysis (IA) were to evaluate the impact of ruxolitinib treatment on symptoms (Myeloproliferative Neoplasm‐10 Total Symptom Score [MPN‐10 TSS]), health‐related QoL (HRQoL; EuroQol‐5 dimension‐5 level [EQ‐5D‐5L] Visual Analog Scale [VAS] score), efficacy outcomes (spleen size and OS), and safety in the IPSS Int‐1‐risk population group who completed the first 12 months of follow‐up or prematurely discontinued the ROMEI study.

## 2 Methods

### 2.1 Study Design, Participants, and Endpoints

ROMEI is an ongoing, national, multicentric, observational, prospective study conducted across 51 centers in Italy that enrolled 508 adult patients (between April 2017 and May 2022) diagnosed with primary or secondary MF, naive to ruxolitinib, starting ruxolitinib as per clinical practice and according to the approved label [12, 14]. Follow‐up was conducted for up to 5 years. This IA focused on clinical features and outcomes of IPSS Int‐1 subgroup patients with MF who completed the first 12 months of follow‐up or prematurely discontinued the study by December 31, 2022, and who did not have missing values for MPN‐10 TSS and for EQ‐5D‐5L at the first visit and took at least one dose of the study medication. The primary endpoint was to assess symptom response (MPN‐10 TSS score) and QoL (EQ‐5D‐5L score). The secondary endpoints included spleen size reduction from baseline, proportion of patients achieving a spleen response, and OS rates. Spleen response according to the International Working Group Myeloproliferative Neoplasms Research and Treatment (IWG MRT) criteria was defined as baseline splenomegaly that was palpable at a length of 5–10 cm and became non‐palpable, or baseline splenomegaly that was palpable at > 10 cm and decreased by ≥ 50%. Patients with a baseline spleen length of < 5 cm were not eligible for spleen response assessments.

Ruxolitinib starting dosages, changes, temporary interruptions, and permanent discontinuation were analyzed. Adverse events (AEs) were graded according to the Common Terminology Criteria for Adverse Events (CTCAE), version 5.0. This study was conducted in accordance with the principles of the Declaration of Helsinki and Good Clinical Practice guidelines of the International Council for Harmonization. The study protocol was reviewed by the independent ethics committee or institutional review board at each center. All study participants provided informed consent before data collection.

### 2.2 Statistical Analysis

Categorical variables are presented as proportions and continuous variables as means (standard deviation; SD) or medians (Q1‐Q3), as appropriate. The proportion of patients with symptom response was evaluated and summarized at each visit until visit 7, focusing mainly on 24 (visit 5) and 48 (visit 7) weeks according to the last observation carried forward (LOCF) approach. HRQoL was analyzed according to the LOCF approach in terms of percentages for each answer level of each EQ‐5D‐5L dimension at baseline, 24 weeks, and 48 weeks and in terms of EQ VAS at each visit until visit 7. The proportion of patients with spleen response was evaluated and summarized at each visit, especially at 24 and 48 weeks. All statistical tables, listings, and analyses were produced using SAS release 9.4 (64‐bit) or later (SAS Institute Inc., Cary, NC, USA).

Further definitions and details are available in Supporting Information S1: file.

## 3 Results

### 3.1 Patient Disposition

**FIGURE 1:**
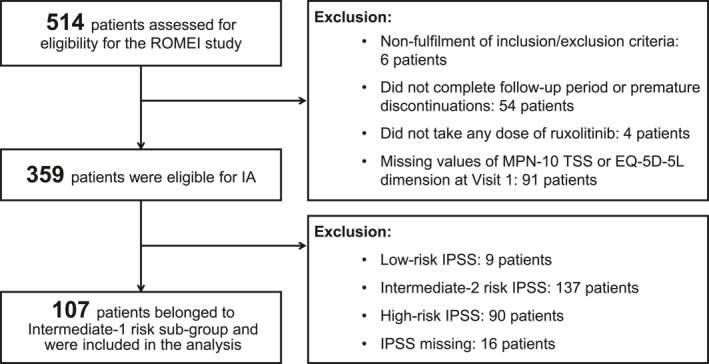
Patient disposition for the IA of the ROMEI study [27]. This IA was performed considering enrolled patients who completed the first 12 months of follow‐up (i.e., patients who completed visit at week 48 [visit 7] or later or patients who discontinued ruxolitinib treatment before but are still under observation) or prematurely discontinued the study by December 31, 2022 (i.e., cutoff date of the present interim analysis). EQ‐5D‐5L, EuroQol 5‐dimensions 5‐levels; IA, interim analysis; IPSS; international prognostic scoring system; MPN‐10 TSS, myeloproliferative neoplasm‐10 total symptom score.

At the data cutoff date (December 31, 2022), 454 of 514 patients screened were enrolled for the IA (i.e., all screened patients who fulfilled the inclusion or exclusion criteria [summary of product characteristics requirements] and who completed the first 12 months of follow‐up [i.e., week 48] or prematurely discontinued treatment or the study earlier; Figure 1). Among the 359 eligible patients, 107 were at Int‐1 IPSS risk.

### 3.2 Demographics and Baseline Characteristics

The mean (± SD) age of patients was 63 (± 12.3) years, and 46 patients were > 65 years old. Despite the relatively younger patient population, 86.9% of patients had a medical history of at least two concomitant diseases. The baseline characteristics of patients are summarized in Table 1.

**TABLE 1:** Demographic and clinical baseline characteristics of the intermediate‐1‐risk population.

	Intermediate‐1‐risk (*N* = 107)
Age, mean ± SD	63 ± 12.3
Age classes, *n* (%)	
≤ 65 years	61 (57.0)
> 65 years	46 (43.0)
Sex, *n* (%)	
Male	56 (52.3)
Female	51 (47.7)
Caucasian race, *n* (%)	107 (100.0)
MF disease, *n* (%)	
Primary MF	59 (55.1)
Post‐PV‐MF	17 (15.9)
Post‐ET‐MF	31 (29.0)
MF disease class, *n* (%)	
Primary MF	59 (55.1)
Secondary MF	48 (44.9)
Time from MF diagnosis to ruxolitinib initiation (months), median (Q1; Q3)	6.5 (1.9; 49.2)
Time from MF diagnosis to ruxolitinib start classes, *n* (%)	
< 12 months	65 (60.8)
≤ 12 months to < 24 months	6 (5.6)
≥ 24 months	36 (33.6)
Bone marrow biopsy available, *n* (%)^a^	101 (94.4)
Grade 0	5 (5.0)
Grade 1	31 (30.7)
Grade 2	42 (41.6)
Grade 3	23 (22.8)
MPN 10‐TSS, mean (SD)	28.3 (18.4)
MPN 10‐TSS class, *n* (%)	
MPN 10‐TSS < 20	38 (35.5)
20 ≤ MPN 10‐TSS ≤ 40	46 (43.0)
MPN 10‐TSS > 40	23 (21.5)
Prior splenic irradiation performed, *n* (%)	1 (0.9)
Manual palpation of the spleen performed, *n* (%)	100 (93.5)
Spleen length (cm), median (Q1; Q3)	6.0 (4.0; 10.0)
Spleen length class, *n* (%)^b^ ^,^ ^c^	
≥ 5 cm	74 (74.00)
≥ 10 cm	27 (27.0)
Prior transfusions performed (PRBC), *n* (%)	
No	98 (91.6)
Yes	9 (8.4)
Hemoglobin at baseline available (g/dL), median (Q1; Q3)	12.3 (10.7; 13.6)
Hemoglobin at baseline (g/L) class 1, *n* (%)	
< 8 g/dL	4 (3.7)
8 to < 10 g/dl	9 (8.4)
10–12 g/dL	33 (30.8)
> 12 g/dL	52 (48.6)
Not available	9 (8.4)
Platelets at baseline (10^9^/L), median (Q1; Q3)	364.0 (217.0; 506.0)
Platelets at baseline (10^9^/L) class < 50 × 10^9^/L	2 (1.9)
50 × 10^9^/L to < 75 × 10^9^/L	2 (1.9)
75 × 10^9^/L to < 100 × 10^9^/L	5 (4.7)
100 × 10^9^/L to 200 × 10^9^/L	11 (10.3)
> 200 × 10^9^/L	77 (72.0)
Not available	10 (9.4)
Disease history by IPSS for the eligible population, MedDRA system organ class > 10%^d^	
Vascular disorders	42 (39.3)
Hypertension	39 (36.5)
Surgical and medical procedures	25 (23.4)
Metabolism and nutrition disorders	23 (21.5)
Gastrointestinal disorders	19 (17.8)
Blood and lymphatic system disorders	18 (16.8)
Neoplasms benign, malignant, and unspecified (incl. cysts and polyps)	15 (14.0)
Cardiac disorders	13 (12.2)
Infections and infestations	12 (11.2)
Reproductive system and breast disorders	11 (10.3)

*Note:* Percentages were computed on patients belonging to the eligible population.

Abbreviations: MF, myelofibrosis; MPN‐10 TSS, myeloproliferative neoplasm‐10 total symptom score; SD, standard deviation.

^a^ Percentages were computed on patients belonging to the eligible population whose bone marrow biopsy was available.

^b^ Percentages were computed on patients belonging to the eligible population who performed manual palpation of the spleen.

^c^ For patients who reported “spleen palpable” equal to “No,” the spleen length was imputed to 0 cm.

^d^ The patient could report more than one medical condition. A patient with multiple occurrences of medical condition within a system organ class (SOC) or preferred term (PT), was counted only once in the SOC total row or PT category or Ongoing status. Terms are coded using MedDRA (Medical Dictionary for Regulatory Activities), version 25.1. Percentages were computed on the eligible population.

### 3.3 Efficacy

#### 3.3.1 Symptoms Response

**FIGURE 2:**
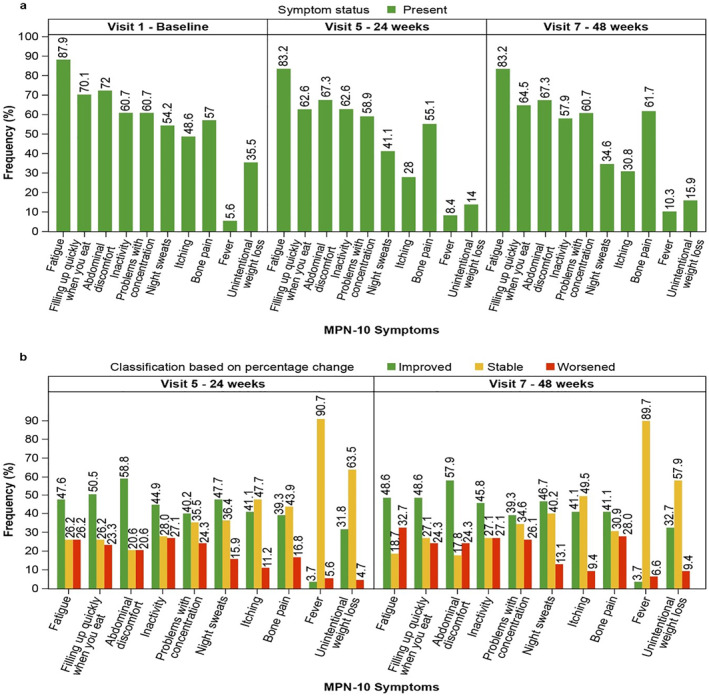
Change in MPN‐10 symptoms from baseline at 24 weeks (visit 5) and 48 weeks (visit 7) in the patient population; (a): Proportion of patients according to MPN 10 symptom status (present); (b). Proportion of patients (improved/stable/worsened) according to the percentage change of MPN‐10 symptoms. The LOCF approach is applied for each symptom; *N* = 107 was used. In case the baseline value is equal to 0, then percentage change was computed as post‐baseline value*100. Patients were classified as ‘improved’ if the percentage change was < 0, ‘stable’ if the percentage change was equal to 0 and ‘worsened’ if the percentage change was > 0. MPN 10; Myeloproliferative Neoplasm 10; LOCF; last observation carried forward.

The mean MPN‐10 TSS at baseline was 28.3 ± 18.4 (min; max: 0; 76). At weeks 24 and 48 respectively, the mean score decreased to 18.2 ± 14.6 (min; max: 0; 60) and 18.5 ± 15.0 (min; max: 0; 61), indicating a mean absolute change from baseline of −10.1 ± 16.7 and −9.8 ± 17.5. The proportion of patients with an MPN‐10 TSS of < 20 was 35.5% at baseline, which increased to 59.8% at both 24 weeks (visit 5) and 48 weeks (visit 7). The distribution of patients according to each symptom present at baseline and at the subsequent time point (24 and 48 weeks) is presented in Figure 2a, and the proportion of patients demonstrating a change in MPN‐10 symptoms is shown in Figure 2b. All symptoms decreased with regard to baseline (visit 1) at 24 and 48 weeks, and most patients improved or remained stable at both 24 and 48 weeks for all symptoms.

The proportion of patients reaching symptoms response was 42.1% at 24 weeks and 43.9% at 48 weeks. Within week 48, 71 patients (66.4%) had a symptoms response, and the median time to first symptoms response was 3.8 months (95% confidence interval [CI]: 2.0–6.7). Among patients who had at least one symptom response based on the IWG‐MRT criteria, 35 (49.3%) lost the first symptoms response, whereas the remaining 36 (50.7%) maintained the first symptoms response within 48 weeks, and the median duration of the first symptoms response was 7.0 months (95% CI: 3.3–11.1).

#### 3.3.2 HRQoL Analysis

**FIGURE 3:**
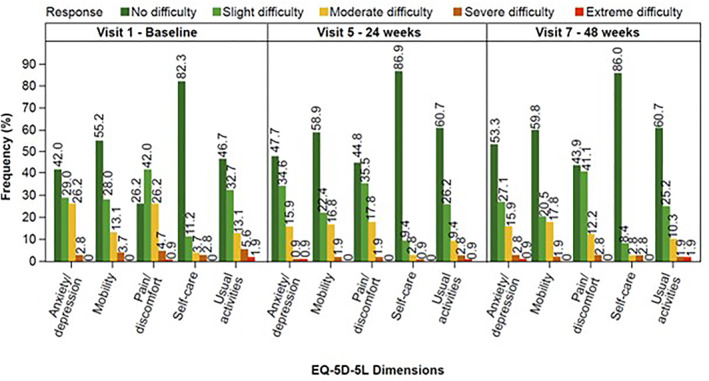
EQ‐5D‐5L questionnaire outcome at baseline, 24 weeks (visit 5), and 48 weeks (visit 7). EQ‐5D‐5L, EuroQol 5‐dimensions 5‐levels.

**FIGURE 4:**
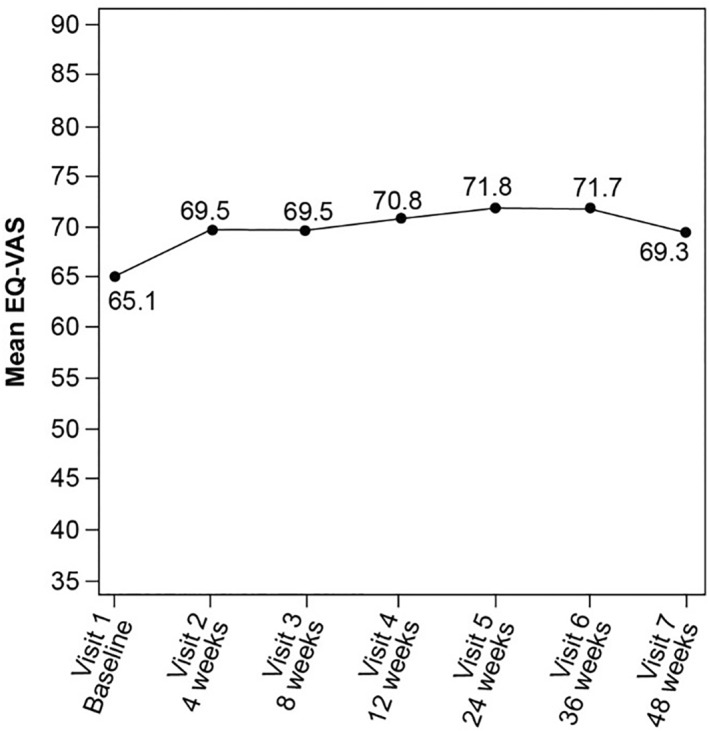
EQ‐5D‐5L questionnaire—Mean EQ‐VAS. The last observation carried forward approach was used to impute missing values. VAS, visual analog scale.

When considering the five dimensions, a higher proportion of patients reported no difficulty at both 24 and 48 weeks compared with that at baseline for each dimension (Figure 3). This finding was consistent with an upward trend observed in the mean EQ‐VAS values (representing patients' health status from worst [0] to best [100]) until 48 weeks, indicating health improvements (Figure 4).

#### 3.3.3 Spleen Response Based on the IWG‐MRT Criteria

Among the patients evaluable for spleen response at 24 and 48 weeks, that is, patients with available spleen measure performed both at baseline and 24 weeks (*N* = 54) and at baseline and 48 weeks (*N* = 47), the spleen response rates were 38.9% (95% CI: 25.9–53.1) and 46.8% (95% CI: 32.1–61.9), respectively.

Considering all spleen evaluations up until the specified cutoff date of the current IA (December 31, 2022), 74 patients (69.2%) were deemed eligible for spleen response (i.e., patients with available spleen measure performed at baseline and at least at one post‐baseline timepoint until the cutoff date). Among these patients, 40 (54.0%) exhibited the first spleen response within the given timeframe, and the median time to achieve the spleen response was 7.4 months (95% CI: 5.3–14.0; Supporting Information S1: Figure S1 and Table S1), and 18 (45.0%) patients experienced loss of the first spleen response within the specified cutoff date. It is important to mention that patients may experience multiple spleen responses. Additional information about the duration of spleen response following the IWG‐MRT criteria is provided in Supporting Information S1: Figure S2 and Table S2.

#### 3.3.4 Overall Survival

Among the 107 eligible patients, 14 (13.1%) died within the cutoff date. Five patients died due to disease progression, while two died due to sudden death during sleep, one patient each on account of cerebral bleeding due to accidental fall, pneumonia, acute myocardial infarction, decay of the general condition, heart failure, SARS‐CoV‐2 pneumonia, and unknown cause of death. Patients who died due to disease progression had initiated ruxolitinib treatment ≥ 12 months after diagnosis.

Median survival time was not estimable. Mean follow‐up duration was 1.9 ± 1.2 years (min; max: 0.2; 4.7 years). At the data cut‐off, 6 patients had less than 12 months follow‐up without treatment discontinuation. More details are provided in Supporting Information S1: Figure S3 and Table S3.

### 3.4 Safety—Dosing Pattern

Overall, 56 patients (52.3%) initiated treatment with a dose of 20 mg twice daily (bid), whereas 13 (12.1%) initiated ruxolitinib therapy at a dose of < 10 mg bid (Supporting Information S1: Table S4). On the basis of baseline platelet counts, 36 patients (33.6%) began initial treatment with a ruxolitinib dose that was lower than expected, 56 (52.3%) received the expected starting dose, 3 (2.8%) received a higher dose than expected, and the remaining 12 (11.2%) could not be classified. The mean daily dose (mg/day) in the first month of treatment was slightly higher, followed by a dose decrease, stabilizing with only small fluctuations from month 2 to month 12 (Supporting Information S1: Figure S4). Within the cutoff date, 76.6% of patients (*n* = 82) had at least one dose adjustment. The proportion of patients with at least one dose increase and one dose decrease was 44.9% (*n* = 48) and 64.5% (*n* = 69), respectively. The mean number of dose adjustments was 2.6 ± 3.3 (min; max: 0; 15).

Fifteen temporary interruptions (*n* = 12, 11.2%) were reported within the cutoff date. Most of these interruptions (*n* = 12, 80%) occurred due to AEs or anomalies detected in laboratory tests. Further details are provided in Supporting Information S1: Table S5.

Twenty‐seven patients (25.2%) discontinued treatment within the cutoff date. The main reasons for discontinuation (Supporting Information S1: Table S6) were death in 8 patients (29.6%), followed by allogeneic stem cell marrow transplantation in 5 patients (18.5%). Patients were followed up for survival even after treatment discontinuation. Of the 14 patients who died within the cutoff date, 6 initially discontinued the treatment for other reasons and then died during observation in the ROMEI study. According to Kaplan‐Meier analysis of time to permanent discontinuation, the median time to discontinuation was not estimable.

Of the 107 patients investigated, 81 (75.7%) reported at least one AE (Table 2). Further details on AEs are summarized in Supporting Information S1: Table S7. Ruxolitinib was generally well tolerated, with an AE profile consistent with that of the overall population in the ROMEI study [13] and that previously reported [8, 15, 16].

**TABLE 2:** Number of patients with adverse events (any grade) by system organ class and preferred term (> 10% patients in any group).

Adverse events by SOC/PT reported within the cutoff date	Intermediate‐1‐risk (*N* = 107) n (%)
Patients with adverse events	81 (75.7)
Blood and lymphatic system disorders	43 (40.2)
Anemia	34 (31.8)
Thrombocytopenia	20 (18.7)
Gastrointestinal disorders	17 (15.9)
General disorders and administration site conditions	25 (23.4)
Asthenia	13 (12.2)
Infections and infestations	28 (26.2)
COVID‐19	12 (11.2)
Investigations	17 (15.9)
Nervous system disorders	13 (12.2)
Respiratory, thoracic, and mediastinal disorders	11 (10.3)
Skin and subcutaneous tissue disorders	13 (12.2)

## 4 Discussion

The worldwide acceptance of ruxolitinib has greatly transformed the treatment landscape for MF. Research from United States (US) community oncology practices revealed that approximately 29% of patients with intermediate‐risk MF are being treated with ruxolitinib as first‐line therapy [17]. In a recent retrospective analysis that aimed to identify risk factors in a large cohort of patients with MF from the TriNETX database, approximately 41% of patients assigned to simplified IPSS groups belonged to the Simplified IPSS 1 point category [18]. Another retrospective review of 491 patients with MF from 45 US community hematology/oncology practices in the Cardinal Health Oncology Provider Extended Network (OPEN) indicated that 58.3% of patients who were assigned a prognostic score belonged to the intermediate risk category as assigned by the physicians at diagnosis [17]. Patients with a lower risk of MF have a considerable disease burden in terms of clinical manifestations, which are not taken into consideration as per the prognostic scoring methods but hamper both QoL and survival, requiring treatment [16].

Studies establishing the safety and efficacy of ruxolitinib treatment in Int‐1‐risk patients with MF are limited [7, 10, 16, 19], and real‐world studies are mainly derived from retrospective observational data collections [9, 10, 19]. Conversely, ROMEI is a prospective clinical study that collected and described clinical features and outcomes in patients with IPSS Int‐I‐risk MF who received ruxolitinib in the clinical practice setting in Italy. Notably, the 107 eligible patients represent approximately 30% of the total population included in the study, underscoring again how Int‐1‐risk patients are very frequently treated with ruxolitinib, and constitute a population of extreme interest because they are potentially projected to have a long exposure to the drug, given the generally more favorable prognosis than patients at higher risk. The baseline characteristics of patients of the ROMEI IPSS Int‐1 cohort are consistent with those reported previously [16]. A recently published retrospective observational study conducted in the real‐world setting of Italy on a large cohort of patients with Int‐1‐risk MF (RUX‐MF study) highlighted that approximately 40% of them possessed large splenomegaly and a high symptom score [10]. Our findings indicated that 64.5% of patients had a symptom score of > 20% and 74% and 27% of patients reported spleen lengths of ≥ 5 cm and ≥ 10 cm, respectively, with approximately 60% of patients reporting grade ≥ 2 bone marrow fibrosis.

This IA demonstrated that ruxolitinib treatment led to a significant reduction in the MPN10‐TSS total score, indicating improvement. The proportion of patients reaching symptoms response at 24 weeks was 42.1%, and that at 48 weeks was 43.9% as compared to that in RUX‐MF study reported earlier (67.9% at 24 weeks) [10], probably due to a greater proportion of patients taking a high starting dose in accordance with the dose‐effect correlation, which was also reported in previous studies [16, 19, 20]. At both 24 and 48 weeks, all symptoms had decreased from the baseline measures, with most patients showing improvement or stability in all symptoms at these intervals. This outcome is supported by findings of the JUMP trial (30%–40% of patients with symptoms response in the IPSS‐1 population) [11] and other previous reports on the treatment of low‐Int‐1‐risk patients, which concluded that ruxolitinib is also beneficial in this subset of patients in the real‐world setting [21, 22].

Ruxolitinib treatment also led to a high rate of spleen response based on the IWG‐MRT criteria, consistent with those observed in the COMFORT‐1 [3] and COMFORT‐2 [23] trials (41.9% and 32.0% of patients demonstrating a spleen volume reduction of ≥ 35%, respectively) and independent studies in patients with IPSS‐1 [16, 24]. At week 24 of the present study, a superior spleen response (38.9%), as compared to that reported in the RUX‐MF study (26.8% [135 of 503], *N* = 595) was noted, while a lower spleen response, as compared to that reported in the JUMP trial (63.8%), was observed [10, 11]. The JUMP trial reported a higher spleen response in the IPSS‐1 category at any timepoint in patients who started ruxolitinib treatment at the 20 mg bid dose (65.0%) and as compared to the 15 mg bid dose (23.9%) [11], which could possibly be linked to the greater proportion of patients on a high dose. Of note, approximately one‐third of patients (33.6%) in the present study started ruxolitinib at a lower‐than‐expected dose, possibly affecting the clinical outcome. A correlation between dose and response has been observed in a prospective Phase 1/2 trial [2] and real‐world studies [25, 26]. As documented in a recent real‐world study conducted on AIFA (Italian Health Authority) monitoring registries in Italy, appropriate starting doses are key to achieve optimal patient outcomes [25]. In a previously published IA of the ROMEI study, Breccia et al. observed that regardless of the IPSS status, a higher spleen response rate (50.0% and 57.7% at 24 and 48 weeks respectively) was achieved by patients receiving starting doses as expected based on the baseline platelet counts versus those starting at lower doses (30.2% and 45.8% at 24 and 48 weeks respectively). Moreover, the median time to first spleen response was shorter in the group receiving recommended starting doses (3.3 vs. 11.1 months; *p* = 0.019). Initiation at the recommended doses also contributed to maximizing the OS (estimated median OS not estimable in recommended starting dose group vs. 4.7 years in the lower than recommended starting dose group, *p* = 0.014) [27]. In our study, 27% of patients had a spleen length of ≥ 10 cm (a potential predictor of a lower spleen response rate) [26], and the proportion was much lesser than that in the RUX MF study (approximately 40%) [10], thus suggesting that treatment for MF in early clinical stages enables better clinical outcomes.

The present study also evaluated the effect of ruxolitinib on the overall QoL, providing, for the first time, fundamental information on the impact of ruxolitinib in this specific cohort. Considering the five dimensions of HRQoL scores, a higher proportion of patients reported no difficulty at both 24 and 48 weeks, compared with baseline, for each dimension that reflected improved QoL. These observations are consistent with those of global health status and improved QoL compared with the standard treatment [28], strongly establishing the positive outcomes of ruxolitinib therapy in the low‐risk population largely comprising patients aged ≤ 65 years (57.0%) who maybe projected for prolonged treatment.

Prior studies, from both controlled clinical studies (COMFORT and JUMP) and real‐world registries (ERNEST), have indicated the beneficial effects of ruxolitinib therapy on OS [3, 8, 29, 30]. In this IA, median survival time was not estimable for Int‐1‐risk patients. The follow‐up period spanned from 0.2 to 4.7 years, with an average of 1.9 ± 1.2 years. The relative short median follow‐up and the described underexposure in approximately 33% patients should be considered when interpreting OS outcome.

This analysis demonstrated that ruxolitinib was generally well tolerated, with an AE profile consistent with that described previously [8, 15, 16], and no new safety concerns were identified. Based on the mechanism of action of ruxolitinib, anemia and thrombocytopenia were the most commonly observed AEs in accordance with previous reports [5]. These events were generally manageable with dose adjustment or temporary interruption, while only few were moderate/severe (Grade ≥ 3) or led to permanent discontinuation, establishing the safety profile in this population.

We also observed a lower proportion of patients (25.2%) with permanent discontinuation as compared to the discontinuation rate reported globally (54.8%) [10]. Our observation is consistent with the discontinuation rate (∼20%) reported in the Int‐1 cohort of the JUMP trial [11].

Overall, responses in Int‐1‐risk patients were more frequent and sustained, consistent with the effects of ruxolitinib reported previously [3, 10, 23, 31].

The prospective study design, which enabled collection of real‐time data and reduced recall bias, provided an accurate representation of patient outcomes. Additionally, the collected data were subject to external monitoring, ensuring the integrity and reliability of the results. However, the study limitations are attributed to its observational design and the relatively small sample size, which was sometimes further reduced due to missing data at certain timepoints (such as spleen measurements or symptoms). Additionally, as for spleen response, the criteria used for evaluating the response, specifically the IWG‐MRT criteria, excluded all patients with a palpable spleen length of < 5 cm below the costal margin.

## 5 Conclusion

This study showed that ruxolitinib treatment led to clinically meaningful reductions in spleen size and symptoms together with a positive impact on OS in IPSS Int‐1‐risk subgroup patients with MF, showing a manageable safety profile and an enhanced HRQoL. This study provides further evidence that administering ruxolitinib earlier in the disease course may be worthy in achieving better outcomes.

## Funding

This study was sponsored by Novartis Farma S.p.A, Milano, Italy.

## Ethics Statement

This study was conducted in accordance with the principles of the Declaration of Helsinki and Good Clinical Practice guidelines of the International Council for Harmonization. The study protocol was reviewed by the independent ethics committee or institutional review board at each center.

## Consent

All study participants provided informed consent before data collection.

## Conflicts of Interest

**Paola Guglielmelli**: honoraria for lectures, presentations, speakers bureaus, manuscript writing, and educational events from Novartis, AbbVie, GSK, and AOP Orphan as well as participation on the advisory board of Novartis, Incyte, and GSK; **Massimo Breccia**: received honoraria from Novartis, Pfizer, Incyte, BMS/Celgene, AbbVie, AOP, and GSK; **Francesco Mendicino**: no conflicts of interest; **Maurizio Martelli**: no conflicts of interest; **Nicola Di Renzo**: no conflicts of interest; **Giuseppe A. Palumbo**: Honoraria for advisory boards/meetings as speaker from AbbVie, AOP Orphan, AstraZeneca, BeiGene, Bristol‐Myers Squibb, GSK, Incyte, MorphoSys, Novartis; support for attending meetings and travel from AbbVie, AOP Orphan, BeiGene, Johnson & Johnson, Novartis, Stemline Menarini; **Monica Crugnola**: no conflicts of interest; **Maurizio Musso:** no conflicts of interest**; Silvia Sibilla**: no conflicts of interest; **Paolo Sportoletti**: no conflicts of interest; **Elisabetta Abruzzese**: no conflicts of interest; **Stefana Impera**: no conflicts of interest; **Alessandra Malato**: no conflicts of interest; **Sergio Siragusa**: no conflicts of interest; **Carmine Selleri**: no conflicts of interest; **Fabrizio Pane**: no conflicts of interest; **Bruno Martino**: no conflicts of interest; **Alessandra Ricco**: no conflicts of interest; **Angelo Gardellini**: no conflicts of interest; **Anna Marina Liberati**: no conflicts of interest; **Agostino Tafuri**: no conflicts of interest; **Maria Langella**: no conflicts of interest; **Marco Brociner**: no conflicts of interest; **Paolo Ditonno**: no conflicts of interest; **Domenico Pastore**: no conflicts of interest; **Olga Mulas:** no conflicts of interest**; Barbara Pocali**: no conflicts of interest; **Marco De Gobbi:** no conflicts of interest; **Erika Morsia**: no conflicts of interest; **Giulia Benevolo**: no conflicts of interest; **Elena Maria Elli**: no conflicts of interest; **Valerio De Stefano**: received honoraria from AbbVie, Bristol Myers Squibb, and Novartis; **Antonietta Pia Falcone**: no conflicts of interest; **Daniele Vallisa**: no conflicts of interest; **Simona Tomassetti**: no conflicts of interest; **Francesca Lunghi**: no conflicts of interest; **Nicola Orofino**: no conflicts of interest; **Giuseppe Carli**: no conflicts of interest; **Tiziana Urbano**: no conflicts of interest; **Alessandro Lucchesi:** no conflicts of interest**; Marta Brunoventre:** no conficts of interest**; Massimiliano Bonifacio**: received honoraria from Novartis, Bristol Myers Squibb, Pfizer, Incyte, Ascentage Pharma, and Amgen; **Gianni Binotto**: received honoraria from Novartis, Bristol Myers Squibb, Incyte, Pfizer, and AOP; **Francesco Cavazzini**: no conflicts of interest; **Paola Ranalli:** no conflicts of interest**; Alessandro Allegra**: no conflicts of interest; **Barbara Anaclerico**: no conflicts of interest; **Serena Mazzotta**: no conflicts of interest; **Filippo Gherlinzoni**: no conflicts of interest; **Mario Tiribelli**: received honoraria from Novartis, Bristol Myers Squibb, Incyte, Pfizer, and GSK; **Chiara Castiglioni**: employees of Novartis Farma S.P.A.; **Marina Landoni**: employees of Novartis Farma S.P.A.; **Diletta Valsecchi**: employees of Novartis Farma S.P.A.; **Michela Magnoli**: employees of OPIS S.r.l.; **Francesco Passamonti**: honoraria for lectures, presentations, speakers’ bureaus, manuscript writing, and educational events from Novartis, Bristol Myers Squibb, AbbVie, GSK, Janssen, and AOP Orphan as well as participation on the advisory board of Novartis, Bristol Myers Squibb/Celgene, GSK, AbbVie, AOP Orphan, Janssen, Karyiopharma, Kyowa Kirin and MEI, Sumitomo, and Kartos; **Francesca Palandri**: consultant and received honoraria from AbbVie, Amgen, AOP, BMS Celgene, Novartis, CTI, GSK, Grifols, Karyopharm, MorphoSys, Sierra Oncology, and Sobi.

## Supporting information


Supporting Information S1

## Data Availability

Novartis is committed to sharing with qualified external researchers, access to patient‐level data and supporting clinical documents from eligible studies. These requests are reviewed and approved by an independent review panel on the basis of scientific merit. All data provided is anonymized to respect the privacy of patients who have participated in the study in line with applicable laws and regulations.
