# Supplementary material for: Clinical Outcomes of Ruxolitinib Treatment in Patients With IPSS Intermediate‐1‐Risk Myelofibrosis: Interim Analysis From an Italian, Prospective Study (ROMEI)
Source: Hematol Oncol. 2026 Mar 25;44(2):e70178. doi: 10.1002/hon.70178 (PMC13014119; doi:10.1002/hon.70178)
Supplement: Supplementary file 1 — Supporting Information S1 [file HON-44-e70178-s001.docx]

**Supplementary Material**

**Clinical outcomes of ruxolitinib treatment in patients with IPSS intermediate-1–risk myelofibrosis: Interim analysis from an Italian, prospective study (ROMEI)**

**Statistical Analysis**

**Definition:**

- Enrolled patients were those who signed a written consent, met the specific criteria for inclusion/exclusion (as stated by the summary of product characteristics), and patients who completed the first 12 months of follow-up (i.e., patients who completed week 48 or later, or patients who discontinued ruxolitinib treatment before but still under observation) or patients who had to stop treatment or prematurely discontinued treatment.
- The myeloproliferative neoplasm total symptom score (MPN-10 TSS) is computed as the sum of the scores in the individual items to achieve a 0-100 score (0 = absent to 100 = worst imaginable). Symptoms response according to the International Working Group Myeloproliferative Neoplasms Research and Treatment (IWG-MRT) criteria was defined as a ≥50% reduction in the MPN-10 TSS from baseline.
- MPN-10 symptoms classification: patients were classified based on percentage changes from baseline at weeks 24 and 28 in the “Improved” (change <0), “Stable” (change=0), and “Worsened” (change >0) categories for each MPN-10 symptom.
- The mean daily dose was computed by dividing the cumulative total dose of ruxolitinib by the number of days in each month (i.e., 4 weeks). In case the patient discontinued treatment (temporarily or permanently), days without treatment were counted considering the dose was equal to 0 in the mean daily dose computation.

**Censoring rules:**

- **Time to first symptoms response** based on International Working Group Myeloproliferative Neoplasms Research and Treatment (IWG-MRT) was calculated as the time between the baseline visit and the first symptoms response for patients who had at least one symptoms response within week 48. For patients without symptoms response within week 48, they were censored considering the visit date of the last MPN-10 assessment effectively performed within week 48.
- **Duration of the first symptoms response** based on the IWG-MRT criteria was calculated only for patients who had at least one symptoms response at week 48. For patients who lost the first symptoms response (i.e., event), it was time from the date of the first response to the date of losing a ≥50% reduction from baseline in the MPN-10 total score within week 48. For patients with the first symptoms response observed within visit 7 (week 48) that was maintained within visit 7 (week 48), it was defined as the time from the date of the first response to the visit date of the last MPN-10 assessment effectively performed within visit 7. Of note, patients who lost the first symptoms response were considered as events even if they reached a new symptoms response within visit 7.
- Patients eligible for spleen response evaluation based on the IWG-MRT criteria were those who performed the manual palpation at both visits and had a baseline splenomegaly that was palpable at ≥5 cm.
- **Time to first spleen response** was the time between the baseline visit and the first spleen response for patients who had at least one spleen response within the cutoff date, i.e., event. For patients without a spleen response within the cutoff date, they were censored considering the visit date of the last manual palpation performed within the cutoff date.
- **Duration of first spleen response** based on the IWG-MRT criteria was calculated only for patients who had at least one symptom response within the cutoff date. For patients who lost the first spleen response (i.e., event), it was time from the date of the first spleen response to the date of losing the spleen response within the cutoff date. For patients with a first spleen response observed that was maintained within the cutoff date, it was defined as the time from the date of the first spleen response to the visit date of the last manual palpation performed within the cutoff date. Of note, patients who lost the first spleen response were considered as having events even if they reached a new spleen response within the cutoff date.
- **Overall survival** considers as event the death due to any cause, and it was defined as the time, in years, from the ruxolitinib start date to the date of death from any cause. Patients who withdrew consent from the study or were lost to follow-up were censored at the time of withdrawal of consent or the last contact date (i.e., end of study), respectively. Patients who were still alive at the cutoff date for the analysis were censored at the cutoff date.
- **Time to permanent discontinuation** was defined as the time, in months, from the first start date of ruxolitinib treatment to the date of permanent discontinuation. Patients who withdrew consent from the study or were lost to follow-up were censored at the time of withdrawal of consent or the last contact date, respectively. Patients who were still in treatment at the cutoff date for the analysis were censored at the cutoff date.
- Kaplan-Meier product limit method was applied to estimate the median time to first symptoms response, duration of first symptoms response, time to first spleen response, duration of first spleen response, median survival time, and median time to permanent discontinuation, along with the discontinuation rate (6-month and 12-month) with 95% confidence interval (CI).
- Data related to ruxolitinib intake are summarized as the mean number of dose adjustments performed, proportions of patients with temporary interruptions and permanent discontinuation, duration of temporary interruption, time to first interruption, and reason for discontinuation.

**Results:**

**Table S1. Time to first spleen response based on the IWG-MRT criteria: summary of events and Kaplan-Meier estimates**

|  | **Intermediate-1-risk**  **(N=107**) |
| --- | --- |
| Patients eligible for at least one spleen response evaluation, n (%) | 74 (69.2) |
| First spleen response based on the IWG-MRT criteria |  |
| Number of events, n (%)^a^ | 40 (54.1) |
| Number of censored, n (%)^a^ | 34 (46.0) |
| Kaplan-Meier estimates (months) |  |
| 25% quartile (95% CI) | 1.5 (1.0, 2.8) |
| Median (95% CI) | 7.4 (5.3, 14.0) |
| 75% quartile (95% CI) | 33.8 (14.0, NE) |

CI, confidence interval; IWG-MRT, International Working Group Myeloproliferative Neoplasms Research and Treatment; NE, not estimable.

Percentages were computed on the eligible population.

^a^Percentages were computed on patients eligible for at least one spleen response evaluation.

Spleen response based on the IWG-MRT criteria was defined as baseline splenomegaly that was palpable at 5-10 cm and became not palpable or baseline splenomegaly that was palpable at >10 cm and decreased by ≥50%. Patients eligible for at least one spleen response evaluation based on the IWG-MRT criteria were patients with manual palpation performed both at baseline and at least at one post-baseline visit. Patients with baseline splenomegaly that is palpable at <5 cm were not eligible for spleen response. Events were defined as the first spleen response observed within the cutoff date. Censored were patients without a spleen response within the cutoff date.

Time to event/censoring was calculated, in months, from the date of visit 1 to the date of the event (i.e., date of the first spleen response) or censoring (i.e., visit date of the last manual palpation performed within the cutoff date).

**Table S2. Duration of the first spleen response based on the IWG-MRT criteria: summary of events and Kaplan-Meier estimates**

|  | **Intermediate-1-risk**  **(N=107)** |
| --- | --- |
| Patients with at least one spleen response (%) | 40 (37.4) |
| Duration of the first spleen response |  |
| Number of events, n (%)^a^ | 18 (45.0) |
| Number of censored, n (%)^a^ | 22 (55.0) |
| Kaplan-Meier estimates (months) |  |
| 25% quartile (95% CI) | 3.0 (1.0, 4.5) |
| Median (95% CI) | NE (3.7, NE) |
| 75% quartile (95% CI) | NE (NE, NE) |

CI, confidence interval; IWG-MRT, International Working Group Myeloproliferative Neoplasms Research and Treatment; NE, not estimable.

.

^a^Percentages were computed on the eligible population with a spleen response based on the IWG-MRT criteria.

Spleen response was defined based on the IWG-MRT criteria as baseline splenomegaly that was palpable at 5-10 cm and became non-palpable or baseline splenomegaly that was palpable at >10 cm and decreased by ≥50%. Baseline splenomegaly that is palpable at <5 cm was not eligible for spleen response. Events were defined as the first spleen response observed and lost within the cutoff date. Censored were patients with the first spleen response observed that is maintained within the cut-off date. Patients who lost the first spleen response were considered. Time to event/censoring was calculated, in months, from the date of the first spleen response to the date of the event (i.e., date of losing the first spleen response) or censoring (i.e., visit date of the last manual palpation performed within the cutoff date).

**Table S3. Overall survival data**

|  | **Intermediate-1-risk**  **(N=107)** |
| --- | --- |
| OS |  |
| Number of events, n (%) | 14 (13.1) |
| Number of censored, n (%) | 93 (86.9) |
| Kaplan-Meier estimates (years) |  |
| 25% quartile (95% CI) | NE (2.07, NE) |
| Median (95% CI) | NE (NE, NE) |
| 75% quartile (95% CI) | NE (NE, NE) |
| Duration of follow-up (years)* |  |
| Mean (SD) | 1.9 (1.2) |
| Median (Q1; Q3) | 1.7 (0.9; 2.1) |
| Range | 0.2- 4.7 |

CI, confidence interval; NE, not estimable; OS, overall survival; SD, standard deviation.

OS event refers to death due to any cause.

Percentages were computed on patients belonging to the eligible population.

Time to event/censoring was calculated, in years, from ruxolitinib start date to the date of the event (i.e., date of death) or date of censoring (i.e., end of study date or cutoff date).

*At data cut-off, 6 patients had less than 12 months follow-up without treatment discontinuation (reasons for study discontinuation being 2 subjects withdrew consent, 1 underwent transplantation, 1 was lost to follow-up, 1 due to physician decision and 1 due to administrative problem).

**Table S4**. **Ruxolitinib initial dose in IPSS Int-1 population**

| **Initial dose** | **Intermediate-1-risk**  **(N=107), n (%)** |
| --- | --- |
| 5 mg/day (2.5 mg bid) | 1 (0.9) |
| 10 mg/day (5 mg bid) | 11 (10.3) |
| 15 mg/day (7.5 mg bid) | 1 (0.9) |
| 20 mg/day (10 mg bid) | 22 (20.6) |
| 30 mg/day (15 mg bid) | 16 (15.0) |
| 40 mg/day (20 mg bid) | 56 (52.3) |

Percentages were computed on patients belonging to the eligible population.

bid, twice a day; IPSS, International Prognostic Scoring System.

**Table S5. Summary of temporary interruptions**

| **Temporary interruptions** | **Intermediate-1-risk**  **(N=107)** |
| --- | --- |
| Total events, n (%) | 15 (100) |
| Resumed treatment at the same dose, n (%) | 7 (46.7) |
| Resumed treatment at a lower dose, n (%) | 7 (46.7) |
| Resumed treatment at a higher dose, n (%) | 1 (6.7) |
| Duration of temporary interruption (days) |  |
| Median (Q1, Q3) | 11 (5, 21) |
| Time to first temporary interruption (days) |  |
| Median (Q1, Q3) | 68.5 (16.5, 238) |

**Table S6. Reasons for treatment discontinuation**

|  | **Intermediate-1-risk**  **(N=107)** |
| --- | --- |
| Patients discontinuing treatment, n (%) | 27 (25.2) |
| Reasons for discontinuation, n (%) |  |
| Death | 8 (29.6) |
| Allo stem cell marrow transplantation | 5 (18.5) |
| AEs | 4 (14.8) |
| Physician decision | 3 (11.1) |
| Withdrew consent | 2 (7.4) |
| Switch to disease-modifying therapy | 2 (7.4) |
| Transformation to acute leukemia | 2 (7.4) |
| Lost to follow-up | 1 (3.7) |

AE, adverse event.

**Table S7. Summary of patients with adverse events by ruxolitinib recommended starting dose group in the eligible population**

| **Adverse events reported within the cutoff date** | **Intermediate-1-risk (N=107), n (%)** |
| --- | --- |
| Patients with adverse events | 81 (75.7) |
| Patients with serious adverse events | 26 (24.3) |
| Patients with drug-related adverse events | 45 (42.1) |
| Patients with drug-related serious adverse events | 7 (6.5) |
| Patients with grade 3 or 4 adverse events | 34 (31.8) |
| Patients with hematologic adverse events | 43 (40.2) |
| Patients with non-hematologic adverse events | 72 (67.3) |
| Patients with anemia of grade 3 with action taken dose  adjusted/temporarily interrupted/permanent discontinued | 5 (4.7) |
| Patients with thrombocytopenia adverse events | 20 (18.7) |

Each patient could experience more than one adverse event. Percentages were computed on patients belonging to the eligible population.

**Figure S1. Reverse Kaplan–Meier plot for time to the first spleen response based on the IWG-MRT criteria**


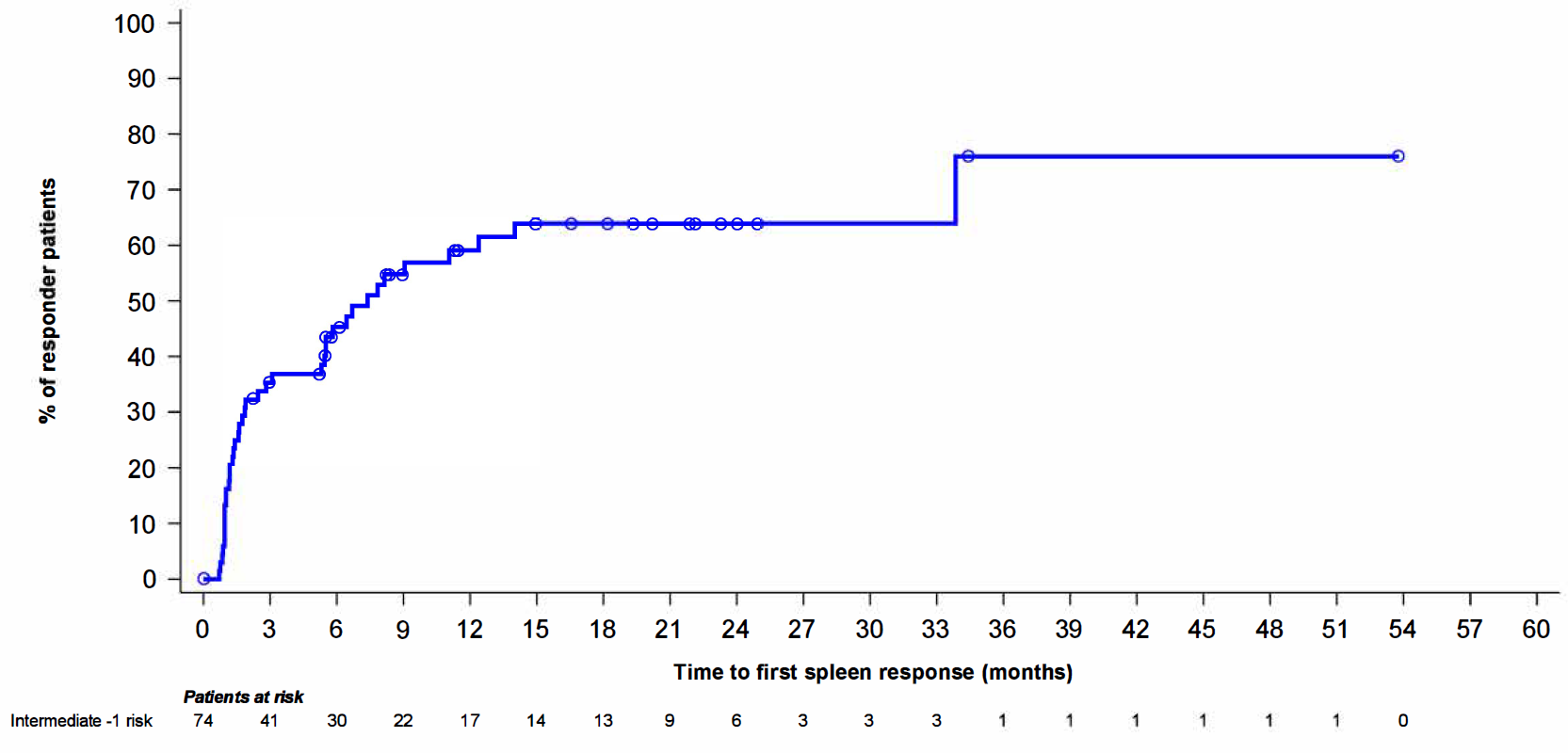


Dots represent censors.

Patients at risk are those eligible for at least one spleen response evaluation (i.e., patients with manual palpation performed both at baseline and at least at one post-baseline visit and without baseline splenomegaly <5 cm) who had no censored observation and did not have spleen response at the considered timepoint yet. Patients with a baseline splenomegaly that is palpable at <5 cm were not eligible for spleen response.

**Figure S2. Kaplan-Meier plot showing the duration of the first spleen response based on the IWG-MRT criteria in IPSS Int-1–risk patients**
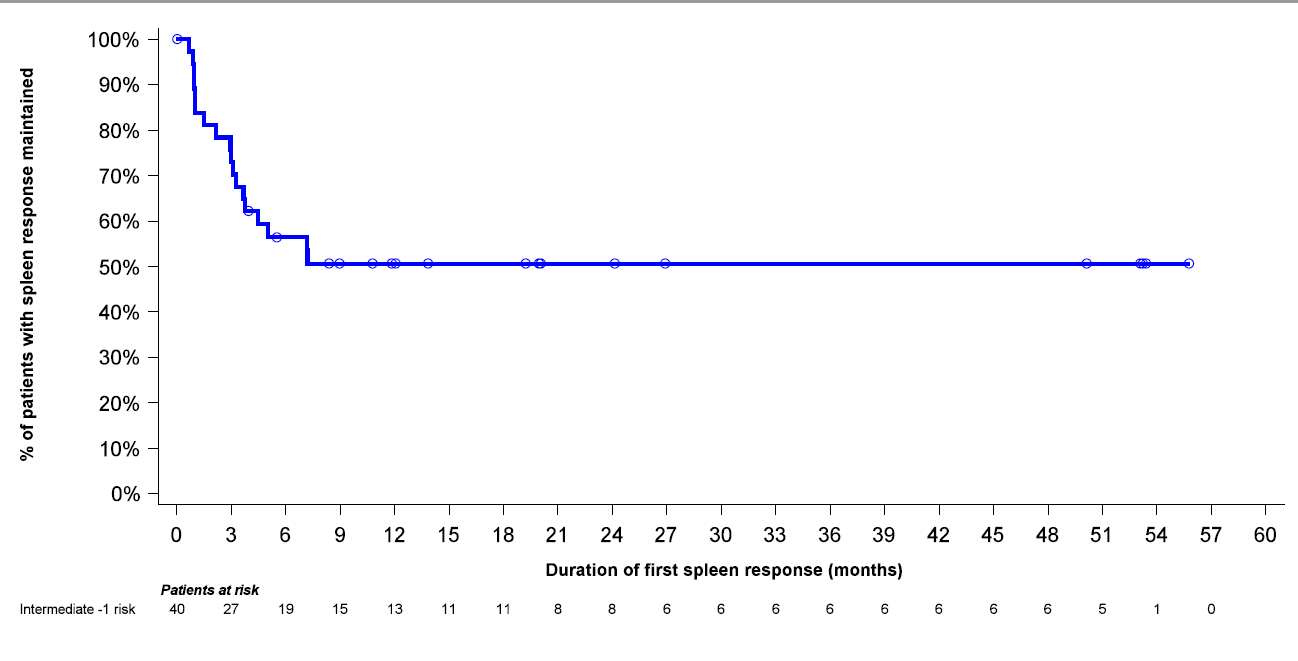


Dots represent censors.

Patients at risk are responders patients who had no censored observation and didn't have spleen response lost at the considered timepoint yet. Only patients with spleen response based on IWG-MRT criteria were considered.

**Figure S3. Overall survival of IPSS Int-1–risk patients with MF**


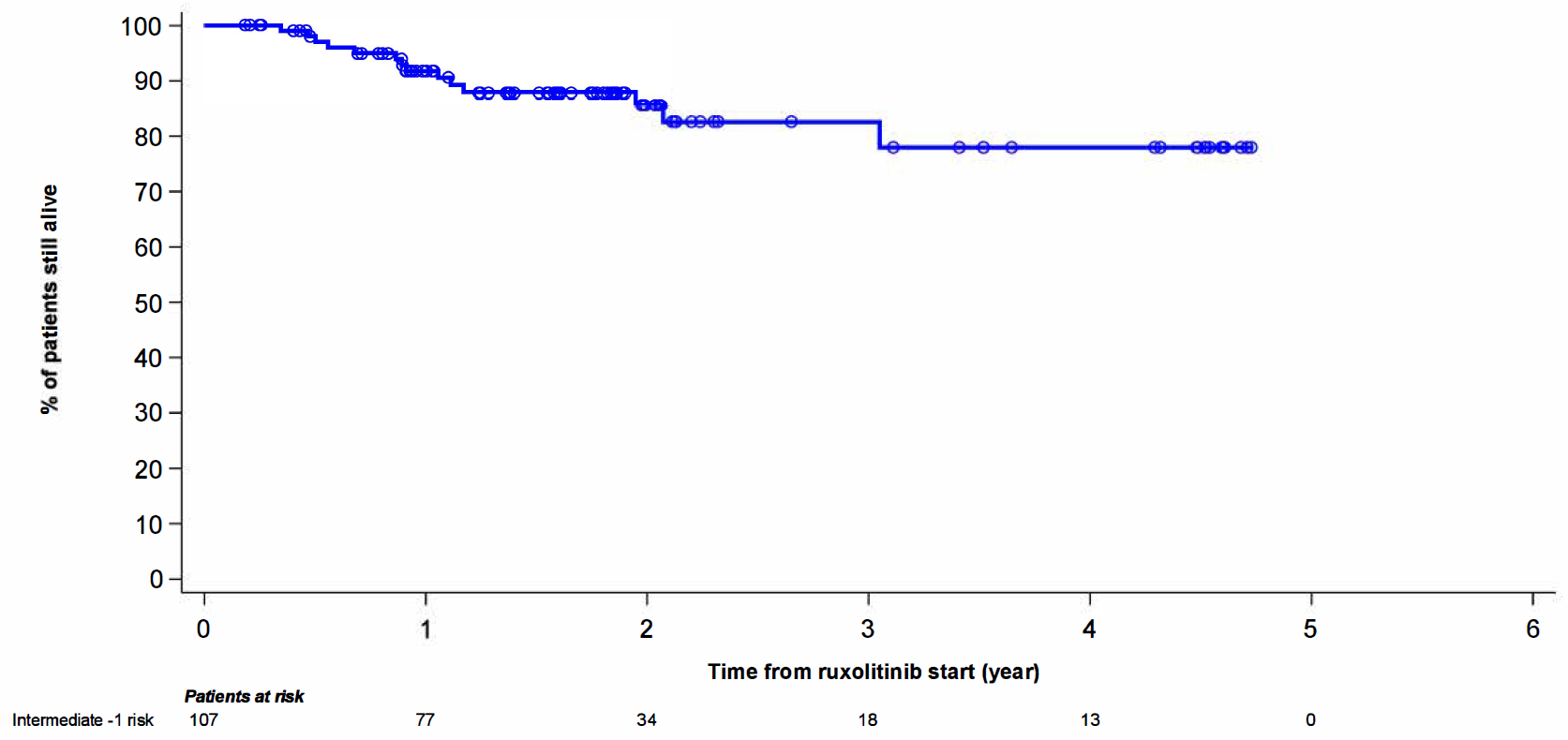


Dots represent censors.

Patients at risk are those who have no censored observation and were still alive at the considered timepoint yet.

**Figure S4. Plot of the mean daily dose of ruxolitinib by month (including all days, including treated or not treated)**


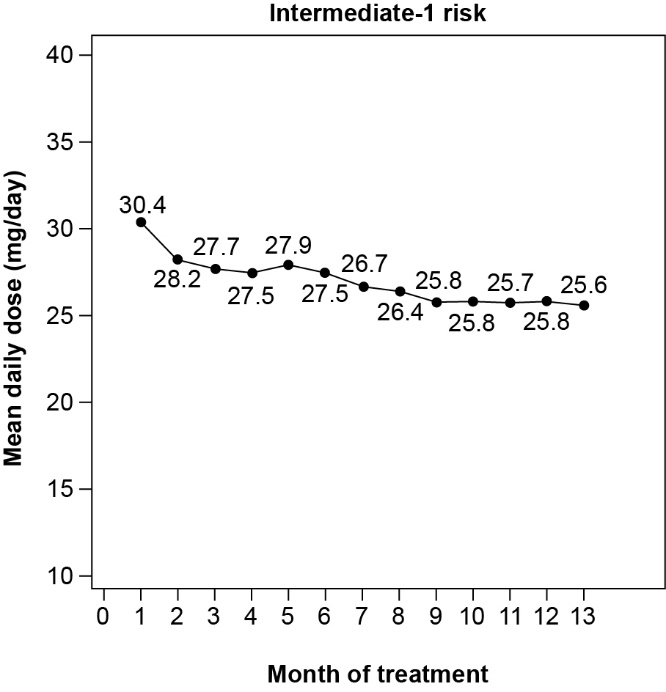


Note: The mean daily dose was computed by dividing the cumulative total dose of ruxolitinib by the number of days in each month (i.e., 4 weeks). If the patient discontinued treatment (temporarily or permanently), then both treated and not treated days were considered for the mean daily dose computation.
